# Tsetse blood-meal sources, endosymbionts and trypanosome-associations in the Maasai Mara National Reserve, a wildlife-human-livestock interface

**DOI:** 10.1371/journal.pntd.0008267

**Published:** 2021-01-06

**Authors:** Edward Edmond Makhulu, Jandouwe Villinger, Vincent Owino Adunga, Maamun M. Jeneby, Edwin Murungi Kimathi, Enock Mararo, Joseph Wang’ang’a Oundo, Ali Abdulahi Musa, Lillian Wambua

**Affiliations:** 1 International Centre of Insect Physiology and Ecology (*icipe*), Nairobi, Kenya; 2 Biochemistry and Molecular Biology Department, Egerton University, Nakuru, Kenya; 3 Institute of Primate Research, National Museums of Kenya, Nairobi, Kenya; 4 Department of Medical Biochemistry, Kisii University, Kisii, Kenya; 5 The Roslin Institute, Easter Bush Campus, University of Edinburgh, Midlothian City, Scotland; 6 School of Biological Sciences, University of Nairobi, Nairobi, Kenya; 7 Department of Medical Laboratory Sciences, Kenyatta University, Nairobi, Kenya; University of California Davis, UNITED STATES

## Abstract

African trypanosomiasis (AT) is a neglected disease of both humans and animals caused by *Trypanosoma* parasites, which are transmitted by obligate hematophagous tsetse flies (*Glossina* spp.). Knowledge on tsetse fly vertebrate hosts and the influence of tsetse endosymbionts on trypanosome presence, especially in wildlife-human-livestock interfaces, is limited. We identified tsetse species, their blood-meal sources, and correlations between endosymbionts and trypanosome presence in tsetse flies from the trypanosome-endemic Maasai Mara National Reserve (MMNR) in Kenya. Among 1167 tsetse flies (1136 *Glossina pallidipes*, 31 *Glossina swynnertoni*) collected from 10 sampling sites, 28 (2.4%) were positive by PCR for trypanosome DNA, most (17/28) being of *Trypanosoma vivax* species. Blood-meal analyses based on high-resolution melting analysis of vertebrate cytochrome c oxidase 1 and cytochrome b gene PCR products (n = 354) identified humans as the most common vertebrate host (37%), followed by hippopotamus (29.1%), African buffalo (26.3%), elephant (3.39%), and giraffe (0.84%). Flies positive for trypanosome DNA had fed on hippopotamus and buffalo. Tsetse flies were more likely to be positive for trypanosomes if they had the *Sodalis glossinidius* endosymbiont (P = 0.0002). These findings point to complex interactions of tsetse flies with trypanosomes, endosymbionts, and diverse vertebrate hosts in wildlife ecosystems such as in the MMNR, which should be considered in control programs. These interactions may contribute to the maintenance of tsetse populations and/or persistent circulation of African trypanosomes. Although the African buffalo is a key reservoir of AT, the higher proportion of hippopotamus blood-meals in flies with trypanosome DNA indicates that other wildlife species may be important in AT transmission. No trypanosomes associated with human disease were identified, but the high proportion of human blood-meals identified are indicative of human African trypanosomiasis risk. Our results add to existing data suggesting that *Sodalis* endosymbionts are associated with increased trypanosome presence in tsetse flies.

## Author summary

Human and animal African trypanosomiasis are neglected tropical diseases with potential to spread to new areas. Wild animals are important reservoirs of African trypanosomes and crucial in the emergence and re-emergence of AT. Vertebrate host-vector-parasite interactions are integral to trypanosome transmission. We identified the diversity of tsetse flies and their trypanosomes, endosymbionts, and vertebrate blood-meal hosts to infer potential transmission dynamics. We found that *Glossina pallidipes* was the major tsetse fly vector and that *Trypanosoma vivax* was the main trypanosome species circulating in the region. Humans, hippopotamus, and buffalo were the most common blood-meal hosts. Buffalo and hippopotamus blood-meals were identified in trypanosome-positive flies. Feeding of the flies on both humans and wildlife may potentiate the risk of the human trypanosomiasis in this ecology. Additionally, we found that the endosymbiont *Sodalis glossinidius* is associated with trypanosome presence in wild tsetse flies. These findings emphasize the importance of understanding the interaction of tsetse flies with their endosymbionts and vertebrate blood-meal hosts in the transmission and control of AT.

## Introduction

African trypanosomes (genus *Trypanosoma*), cyclically transmitted by the tsetse fly vector (genus *Glossina*), cause a group of diseases known as African trypanosomiasis (AT). The disease is called sleeping sickness (human African trypanosomiasis, HAT) in humans and nagana (African animal trypanosomiasis, AAT) in animals. African trypanosomiasis is endemic in regions inhabited by the insect vector in 37 countries in Africa, rendering approximately 70 million people and 60 million cattle in AT-endemic regions at risk of infection [1,2]. Consequently, reduced productivity due to chronic disease in humans and animals and loss of livestock through death threatens food security, quality of living, and economic stability, particularly in regions where pastoralism is the main economic activity [3–5]. Therefore, more effective AT control and management strategies are required.

Control of AT has involved active surveillance, vector control strategies, and mass chemotherapy [6,7]. Notably, chemotherapy has been limited by increasing levels of resistance to the available trypanocides, chemotoxicity, and unavailability of new drugs [6,8]. To address the limitations associated with chemotherapy, disruption of trypanosomes transmission through vector control is crucial. Vector control is largely applied in areas where livestock are kept [9,10]. However, wild animals sustain the life cycles of tsetse flies [11–13] as well as the trypanosomes [14,15] and are thus an important factor in the transmission dynamics of AT, particularly in wildlife ecologies. Tsetse fly blood-meal sources are highly variable, especially in wildlife areas. Hence, one sampling area cannot be used to make generalized conclusions on tsetse feeding behavior [12]. Consequently, identification of tsetse fly blood-meal host sources in specific regions can help to elucidate wildlife species that are potentially involved in AT transmission and provide a baseline for research towards improving vector-control strategies, particularly in wildlife-human-livestock interfaces that serve as hotspots for the emergence and re-emergence of AT.

Transmission of vector-borne pathogens is also highly influenced by vector competence, which is affected by various factors, including vector endosymbionts [16–19]. In the case of AT, *Wigglesworthia glossinidia*, *Sodalis glossinidius*, *Wolbachia pipientis*, and *Spiroplasma* are well-defined tsetse fly endosymbionts [20–22]. *Sodalis* and *Wigglesworthia* have been shown to increase tsetse vector competence [23–27], while *Spiroplasma* may potentially reduce vector competence [19]. Therefore, the influence of endosymbionts on the susceptibility of tsetse flies to trypanosomes is likely to have an impact on disease transmission. Despite numerous studies on the influence of endosymbionts on tsetse fly competence [19,23,25,28–31], studies on the presence and influence of tsetse fly endosymbionts in wildlife-livestock-human interfaces are scant in Kenya.

The Maasai Mara National Reserve (MMNR) is a prime tourist destination in Kenya that is surrounded by a number of ranches and is thus characterized by constant interactions between wildlife and humans and their livestock. With endemic tsetse fly populations, cases of tourists contracting HAT in the MMNR have been reported [32,33]. Therefore, the MMNR is an ideal study site for investigating the contribution of tsetse fly blood-meal sources and the major endosymbionts of tsetse flies in relation to transmission of African trypanosomes in a human-livestock-wildlife interface. We conducted a cross-sectional study to identify trypanosome species circulating in wild-caught tsetse flies from the MMNR and their blood-meal sources. Further, we sought to identify the tsetse endosymbionts, *Sodalis*, *Wolbachia*, *Spiroplasma*, and salivary gland hypertrophy virus in the tsetse flies and their correlations with trypanosome presence.

## Materials and methods

### Ethics statement

Ethical clearance for this research in protected areas was sought from and approved by the Kenya Wildlife Service (KWS) Research Authorization committee.

### Study area

**Fig 1:**
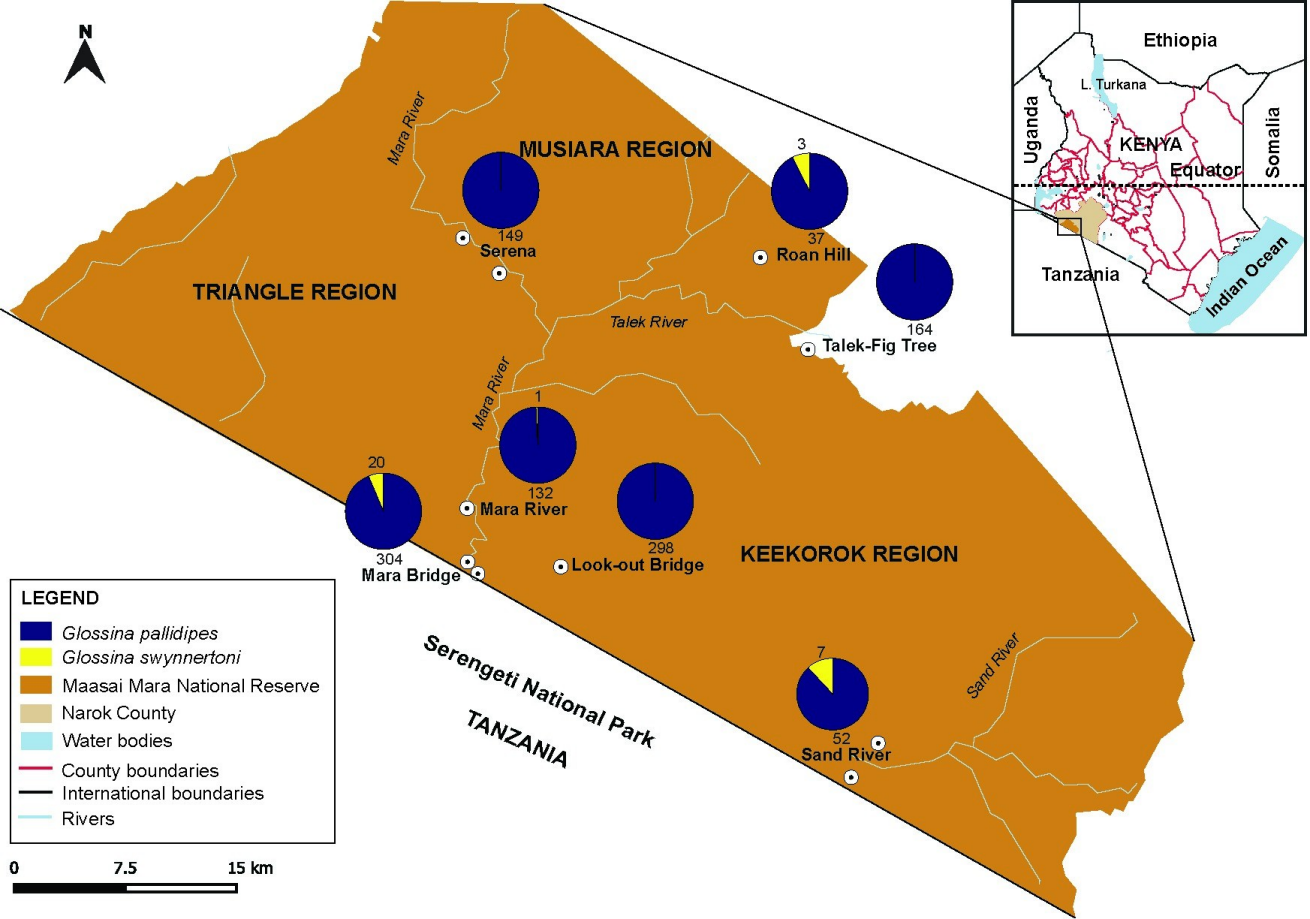
Map of the Maasai Mara National Reserve showing tsetse fly sampling sites and number of tsetse flies sampled per species. Total numbers of flies sampled in each sampling site are indicated in the pie charts. This map is republished with data from the following sources: https://www.wri.org/resources/data-sets/kenya-gis-data from the World Institute Resources, 2007 [34]; https://africaopendata.org/dataset/kenya-counties-shapefile from openAfrica, 2015 [35]; and http://geoportal.rcmrd.org/layers/?limit=100&offset=0 from RCMRD Geoportal, 2016 [36].

Field sampling was performed between June and July 2016, within the MMNR (1°29′24″S 35°8′38″E, 1500 m above sea level), located in southwest region of Kenya, which is contiguous with the Serengeti National Park (SNP) in Tanzania (Fig 1). This sampling site is located approximately 150 km south from the equator and covers an area of 1500 km^2^. The MMNR is home to a diverse variety of flora and fauna and is famously known for its wild animals and the ‘Great Migration’ of wildebeests, zebras, and antelopes across the Mara River. Grassland forms the major vegetation cover in this ecosystem, with swampy grounds found around the riverbanks. The sampling sites were selected along the rivers due to their high populations of animals (Fig 1).

### Tsetse collection and identification

Tsetse flies were trapped at the start of the annual wildebeest migration between June and July 2016 using Nguruman (Ngu) traps baited with acetone and cow urine. Traps were set in the morning (10–11 am) at different sampling sites in the various regions demarcated by Mara, Talek, and Sand Rivers, and at the wildlife crossing points across the Mara River at the border of Kenya and Tanzania’s SNP (Fig 1). The traps were emptied after 24 hours, and trapped flies were transferred into 50-mL falcon tubes and stored in dry ice before transportation in liquid nitrogen to the laboratory at the International Centre of Insect Physiology and Ecology (*icipe*), Nairobi, where they were sorted and stored in a -80°C freezer. The flies were identified to species level under a light microscope (Stemi 2000-C, Zeiss, Oberkochen, Germany) based on standard published taxonomic keys [37]. Tsetse were identified on a cold pack for not more than five minutes during which we removed fly wings and legs. They were then stored at -80°C, awaiting DNA extraction.

### Nucleic acid extraction

Before DNA extraction, individual tsetse flies were surface sterilized by quick submersion in 1% bleach, followed by 70% ethanol for five minutes and rinsing with distilled water. Immediately after surface sterilization, individual flies were homogenized for 20 seconds in a Mini-beadbeater-16 (BioSpecs Inc., Bartlesville, OK, USA) using six 2-mm zirconium beads in 1.5-ml microcentrifuge tubes. DNA was extracted from the homogenate of each sample using the ammonium acetate protein precipitation method described by Adams *et al*. [38], with slight modifications. Briefly, 300 μl of cell lysate buffer (10 mM Tris-HCl, pH 8.0, 0.5% SDS and 5 mM EDTA) was added into homogenized samples and incubated for 90 minutes at 65°C. Thereafter, 100 μl of protein precipitate solution (8M ammonium acetate and 1M EDTA) was added to each mixture, which were vortexed for 30 seconds, incubated on ice for 30 minutes, and centrifuged at 14,000 x g for 15 minutes at 4°C. The supernatants were transferred into new 1.5-ml microcentrifuge tubes containing 300 μl of isopropanol, mixed gently by inverting 100 times, and centrifuged at 14,000 x g for 30 minutes. The supernatants were pipetted off and subsequently, 300 μl of ice-cold 70% molecular grade ethanol was added to each pellet, gently mixed by inversion, and centrifuged at 14,000 x g for 30 minutes. Ethanol was pipetted off and the pellets were air-dried overnight. The DNA pellets were solubilized by adding 100 μl of PCR grade water and quantified using a NanoDrop 2000 Spectrophotometer (Thermo Scientific, NJ, USA). Concentrations were adjusted to 50 ng/μl using PCR grade water.

### PCR identification of African trypanosomes

Trypanosome parasites present in flies were detected using trypanosome-specific ITS1 CF and BR primers (S1 Table) as described by Njiru *et al*. [39]. *Trypanozoon* species were further resolved using species-specific primers (S1 Table), whereby glycosylphosphatidylinositol-phospholipase C polypeptide (GPI-PLC) and serum resistance-associated (SRA) species-specific primers were used to identify *T*. *brucei brucei* and *T*. *brucei rhodesiense*, respectively, by PCR [40]. *Trypanosoma congolense savannah* was identified according to Masiga *et al*. [41].

PCR reactions were carried out in 20-μl reaction volumes containing 10.4 μl of PCR grade water, 1× GeneScript PCR reaction buffer and 1.6 units of Green Taq DNA polymerase enzyme (GeneScript, New Jersey, USA), 1 μl (final concentration 0.5 μM) of each primer, and 200 ng DNA template. The PCRs were performed in a SimpliAmp Thermal Cycler (Applied Biosystems, California, USA) programmed as follows; initial denaturation at 94°C for 3 minutes followed by 30 cycles of denaturation at 94°C for 30 seconds, annealing at temperatures specific for each primer pair (S1 Table) for 30 seconds, and extension at 72°C for 45 seconds, and a final extension at 72°C for 7 minutes. PCR grade water was used as a negative control in place of DNA template. DNA obtained from characterized and archived stocks of African trypanosome species were used as positive controls. The PCR products were size separated by ethidium-stained agarose gel electrophoresis and viewed under UV light.

Gel products of representative samples were purified using QIAquick Gel Extraction Kit (QIAGEN, Valencia, CA) according to the manufacturer’s instructions and sequenced at Macrogen (The Netherlands). The sequences were analyzed and aligned using the MAFFT plugin in Geneious software version 11.1.4 [42]. Trypanosome species were confirmed by sequence alignments with basic local alignment search tool (BLAST) hits [43] with > 99% homology.

### Host blood-meal identification

Blood-meal sources were determined by PCR coupled with high-resolution melting (HRM) analysis of vertebrate cytochrome c oxidase subunit I (COI) and cytochrome b (cyt b) mitochondrial genes as previously described [44–46]. We analyzed 760 flies, representing 65% of the sampled population, including all engorged flies (n = 39), trypanosome-positive flies (n = 28), and randomly selected non-engorged flies. The PCRs were carried out in 20-μl reaction volumes, which included 4 ul of 5× Hot FIREPol EvaGreen HRM Mix (Solis BioDyne, Tartu, Estonia), 0.5 μM of each primer, 50 ng of DNA template, and 10 μl of PCR grade water. The PCR cycling conditions included an initial denaturation at 95°C for 15 minutes followed by 35 cycles of denaturation at 95°C for 30 seconds, annealing at specific temperatures for COI and cyt b primers (S1 Table) for 30 seconds and elongation at 72°C for 30 seconds. This was followed by a final extension at 72°C for 7 minutes. Thereafter, HRM analysis of PCR products was conducted as described by [44–46]. HRM profiles were analyzed using the Rotor-Gene Q software version 2.1 with normalized regions between 76.0–78.0°C and 89.50–90.0°C. Amplicons representative of each unique HRM profile were purified using ExoSAP-IT (USB Corporation, Cleveland, Ohio, USA) according to the manufacturer’s instructions and sequenced at Macrogen. The sequences were analyzed and aligned using the MAFFT plugin in Geneious software version 11.1.4 [42]. Vertebrate species were confirmed by sequence alignments and ≥99% homology with sequences obtained using the BLAST.

### PCR identification of *Sodalis glossinidius*, *Wolbachia*, *Spiroplasma*, and salivary gland hypertrophy virus

We screened all of the sampled tsetse flies for their endosymbionts, *S*. *glossinidius*, *Wolbachia*, *Spiroplasma* and salivary gland hypertrophy virus (SGHV). The endosymbionts were amplified in 20-μl PCR volumes using endosymbiont-specific primers [47–50] (S1 Table) and similar reagent concentrations and thermocycling steps as described above for host blood-meal identification. Positive controls for *Wolbachia*, *Spiroplasma*, and *Sodalis* were obtained from positive samples from our study that were confirmed by sequencing. A plasmid standard from a synthetic construct of the *P74* gene of SGHV from GenScript was used as a positive control. PCR-grade water was used as negative control template. The amplified products were size separated in 2% (W/V) agarose gels. Representative endosymbiont amplicons (S3 Fig) were purified using ExoSAP-IT (USB Corporation) and sequenced for confirmation at Macrogen (The Netherlands).

### Statistical analyses

For deviations from the expected 1:1 sex ratio proportion within tsetse fly species, exact binomial tests with 95% confidence intervals were used. A t-test was used to compare frequencies of host blood-meals between the tsetse fly species. We also tested for correlations between trypanosome presence and specific endosymbionts identified in each of the sampled tsetse species using generalized linear models (GLM). All statistical analyses were conducted within RStudio.

## Results

### Tsetse fly species identified

A total of 1167 tsetse flies were collected from the ten sampling sites, of which 1136 were *G*. *pallidipes* and 31 were *G*. *swynnertoni*. Most of the *G*. *swynnertoni* flies sampled (27/31) were from sites close to the border between the MMNR and the SNP, i.e. Mara Bridge (n = 20/31) and Sand River (n = 7/31) sampling sites (Fig 1). More female than male tsetse flies were sampled for both *G*. *pallidipes* (P = 9.285e-12, 95% CI: [57, 0.63]) and *G*. *swynnertoni* (P = 0.0009, 95% CI: [0.63, 0.93]).

### Trypanosome species identified in sampled tsetse flies

Trypanosome DNA amplified in 28 (2.40%) of the 1167 tsetse flies sampled (Table 1). Of the African trypanosome species identified, 61% were *T*. *vivax* (17/28), 25% were *T*. *congolense savannah* (7/28), and 14.3% were *T*. *brucei brucei* (4/28) (GenBank accessions MK684364-MK684366). We did not detect DNA from more than one trypanosome species in any specimen. Samples positive for trypanosomes by PCR are shown in S1 Fig. Trypanosome presence was higher in *G*. *swynnertoni* (n = 7/31, 22.6%) than in *G*. *pallidipes* (n = 21/1136, 1.8%).

**Table 1:** Trypanosome species detected in *Glossina pallidipes* and *Glossina swynnertoni*.

Tsetse fly species	Number of tsetse flies screened	Trypanosome species		
		*T*. *b*. *brucei*	*T*. *c*. *savannah*	*T*. *vivax*
*G*. *pallidipes*	1136	3	7	11
*G*. *swynnertoni*	31	1	0	6
Totals	1167	4	7	17

### Tsetse blood-meal sources identified

**Fig 2:**
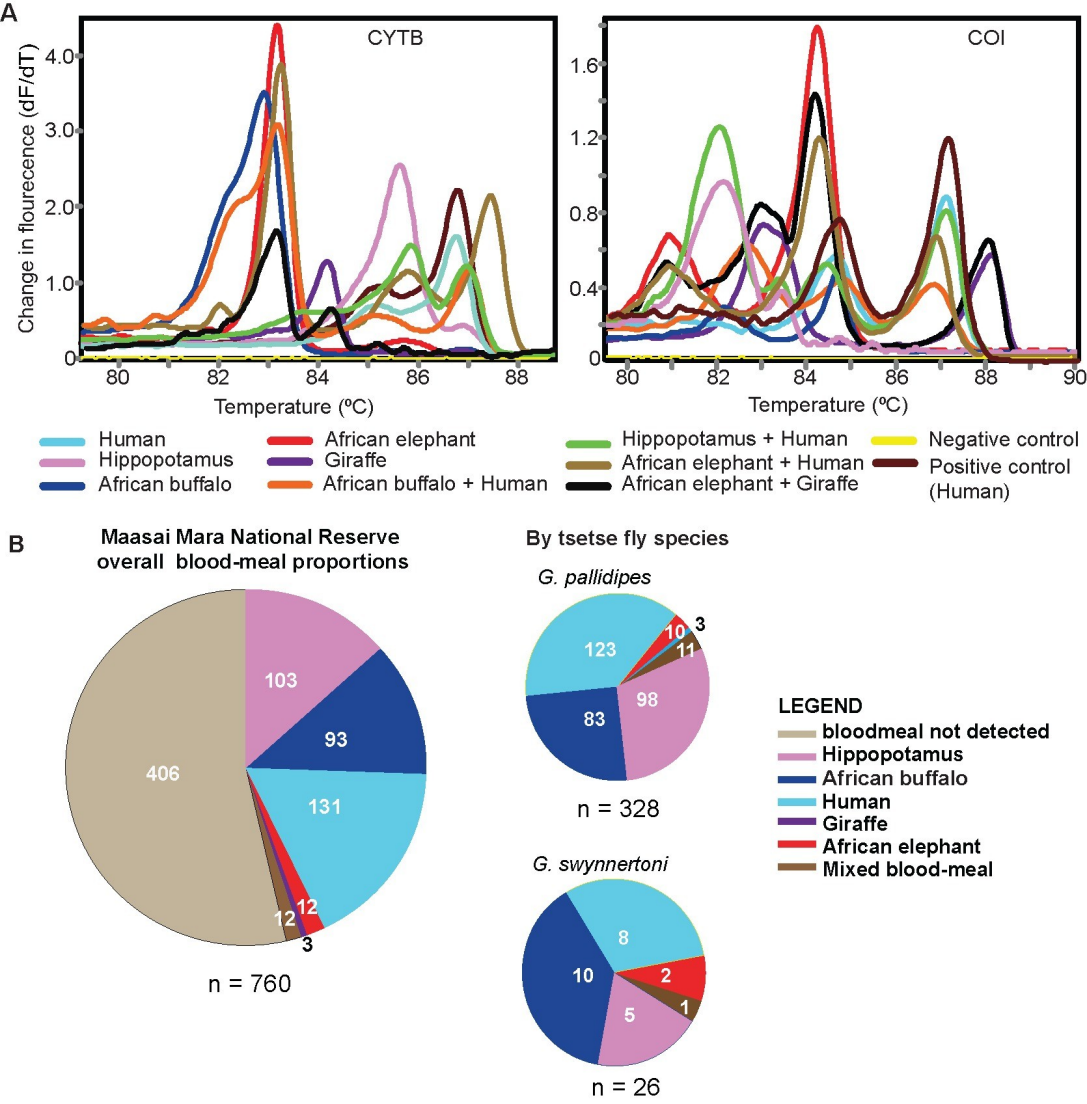
Blood-meal HRM profiles and proportions of vertebrate species identified. Panel **A.** HRM profiles of single species and mixed species blood-meals. Mixed blood-meals were determined by matching melt rate profiles to those of more than one blood-meal control. Panel **B.** Overall and per-tsetse-species proportions of vertebrate blood-meal sources.

Vertebrate blood-meals were detected and identified in 46.6% (354/760) of the tsetse flies analyzed, of which 328 were *G*. *pallidipes* and 26 were *G*. *swynnertoni* (Fig 2 and S2 Table). The most common source of blood-meal was from humans (*Homo sapiens*) (n = 131) (*cyt b* GenBank accession MK684355, MK684357), followed by hippopotamus (*Hippopotamus amphibious*) (*cyt b* GenBank accession MK684356) (n = 103), African buffalo (*Syncerus caffer*) (*cyt b* GenBank accessions MK684354, MK684358) (n = 93), African savannah elephant (*Loxodonta africana*) (*cyt b* GenBank accession MK684359) (n = 12), and giraffe (*Giraffa camelopardis*) (*cyt b* GenBank accession MK684360) (n = 3). There were 406 samples, including six flies with trypanosome DNA, that had HRM peaks lower than 0.5 rate in fluorescence (dF/dT) or no peaks and thus qualified as having no detectable blood-meal traces. The vertebrate blood-meal detection rates were 94.87% and 43.69% in engorged and non-engorged flies, respectively.

Humans were the most frequently identified blood-meal source in *G*. *pallidipes*, whereas African buffalo was the major blood-meal source of *G*. *swynnertoni* (Fig 2B and S2 Table). However, there was no significant difference in the mean blood-meal sources between the two tsetse fly species (t_4_ = 2.47, p = 0.069). Further, we observed that of the 28 tsetse with trypanosome DNA, 14 (10 *G*. *pallidipes* and four *G*. *swynnertoni*) had blood-meals from African buffalo and eight (*G*. *pallidipes*) had blood-meals from hippopotamus.

Twelve mixed blood-meals were detected (Fig 2), accounting for 3.4% of the blood-meals. These samples had distinct melt curves that matched multiple reference samples. Mixed blood-meals were further confirmed by analyzing mixed chromatograms sequenced from representative PCR-HRM amplicons (S2 Fig). Of these, mixed blood-meals from human and buffalo were most frequent (6/12), followed by human and elephant (3/12), elephant and giraffe (2/12), and human and hippopotamus (1/12) blood-meals (Fig 2 and S2 Table).

### Correlations between endosymbionts and presence of African trypanosomes

A total of 77 (n = 1167, 6.6%) flies (74 *G*. *pallidipes*, three *G*. *swynnertoni*) had DNA of the endosymbiont *S*. *glossinidius* (GenBank accessions MK684361-MK684363) (S3 Table.). Notably, a greater proportion of *S*. *glossinidius*-positive *G*. *pallidipes* flies were positive for trypanosomes (7/74, 9.46%) than *G*. *pallidipes* without *Sodalis* endosymbionts (14/1062, 1.32%) (Deviance = 14.205, P = 0.0002; Table 2). Five of the *G*. *pallidipes* that were positive for *Sodalis* had *T*. *congolense* DNA, while two had *T*. *vivax* DNA. In *G*. *swynnertoni*, only one out of three flies with *Sodalis* had trypanosome DNA (*T*. *vivax*), with no association between *Sodalis* and trypanosome presence (Deviance = 0.2023, P = 0.6529; Table 2).

**Table 2:** Statistical correlations of *Sodalis glossinidus*, *Wolbachia*, and *Spiroplasma* endosymbionts with trypanosome DNA in *G*. *pallidipes* and *G*. *swynnertoni*.

	*G*. *pallidipes*			*G*. *swynnertoni*			*G*. *pallidipes*			*G*. *pallidipes*	
	T+	T-		T+	T-		T+	T-		T+	T-
So+	7	67	So+	1	2	W+	1	17	Sp+	0	17
So-	14	1048	So-	6	22	W-	20	1098	Sp-	21	1098
P = 0.0002*			P = 0.6529			P = 0.232			P = 0.5218		

**Abbreviations: So+/So-** *Sodalis* positive/negative, **W+/W-** *Wolbachia* positive/negative, **Sp+/Sp-** *Spiroplasma* positive/negative, **T+/T-** trypanosome positive/negative.

Seventeen out of 1,136 (1.5%) *G*. *pallidipes* were *Spiroplasma*-positive, none of which had detectable trypanosome DNA. Eighteen out of 1136 (1.6%) *G*. *pallidipes* were positive for *Wolbachia* (deposited GenBank accessions MK680053-MK680056) (S3 Table). No *Spiroplasma* or *Wolbachia* were detected in *G*. *swynnertoni*. Only one *G*. *pallidipes* with trypanosome DNA was positive for the *Wolbachia* symbiont. However, there was no significant association between trypanosome presence and *Spiroplasma* (Deviance = 0.5218, P = 0.4701) or *Wolbachia* (Deviance = 1.4284, P = 0.232) in *G*. *pallidipes*. No SGHV was detected in this study.

## Discussion

Transmission of vector-borne diseases is dependent on vector competence and the interactions between vectors and their vertebrate hosts that are reservoirs of the parasites [12,51]. This cross-sectional study revealed that humans, hippopotamus, and African buffaloes were the most frequent blood-meal sources of tsetse flies in the MMNR, a wildlife ecology in Kenya. We also found that the endosymbiont, *S*. *glossinidius*, was positively correlated with trypanosome presence in wild-caught *G*. *pallidipes* tsetse flies in the MMNR, supporting the hypothesis that *Sodalis* potentiates African trypanosome transmission in tsetse flies [25,26,52]. However, we found no correlation between *Wolbachia* and trypanosome presence. Although we only found *Spiroplasma* in *G*. *pallidipes* that did not have trypanosomes, the limited numbers of *G*. *pallidipes* (21, 1.8%) with trypanosome DNA or *Spiroplasma* (17, 1.5%) precluded meaningful analysis of potential *Spiroplasma-*trypanosome correlations. Nevertheless, a recent study in Uganda demonstrated a negative correlation between *Spiroplasma* and *T*. *brucei brucei* in *Glossina fuscipes*, warranting further investigation on whether the same effect would be observed in the MMNR [19]. We found no evidence of SGHV endosymbionts in the tsetse populations analyzed. Taken together, these findings emphasize the importance of understanding the complete spectrum of interactions amongst vertebrates, tsetse fly vectors, endosymbionts, and trypanosome parasites, particularly in the context of wildlife-livestock-human interfaces where emergence and reemergence of AT and other vector-borne diseases are reported.

*Glossina pallidipes* was the most abundant tsetse species sampled in the MMNR in this study, while *G*. *swynnertoni* was less abundant. This finding corroborates previous studies in which these two savannah tsetse species were found to be predominant in the Maasai Mara-Serengeti ecosystem of Kenya and Tanzania [5,53]. As both species are competent vectors of human and animal trypanosomes [52,54,55], their presence highlights the persistent risk of AAT and HAT in the MMNR. *Glossina pallidipes* is a widely-spread species in Kenya and intense control strategies have had limited success [56]. However, populations of *G*. *pallidipes* have been found to be clustered genetically in Kenya [57], necessitating tailor-made control and monitoring strategies for the different clusters for effective tsetse fly eradication. Unlike *G*. *pallidipes*, the geographical range of *G*. *swynnertoni* in Kenya is limited to a narrow belt within the Maasai Mara-Serengeti ecosystem, which has resulted in the prioritization of this tsetse species as a target for elimination in East Africa [58]. Extensive efforts have been employed over the last four decades to reduce *G*. *swynnertoni* populations using various techniques as comprehensively reviewed by Nagagi and co-workers [58]. These have included spraying with both residual and non-residual insecticides, use of mechanical traps and baits with insecticide-impregnated traps or cloth targets, and insecticide-treated animals as live mobile targets. Coordinated studies are needed to evaluate their effect on tsetse populations and quantify their impact in East Africa.

Despite recent cases of HAT (caused by *T*. *b*. *rhodensiense*) being reported in East Africa [59], the trypanosome species identified in this study are only those responsible for causing trypanosomiasis in animals. Kenya is currently classified by the WHO as a country with diminished incidence of HAT (<10 cases in the last decade), with recent cases being reported in tourists returning from the MMNR in 2012 [32,33]. Nevertheless, the persistent presence of *G*. *pallidipes* and *G*. *swynnertoni*, which are competent vectors of *T*. *b*. *rhodensiense*, coupled with the relatively higher incidences of HAT in neighboring Tanzania and Uganda and increased tourism, reinforces the need for coordinated surveillance and diagnosis in the MMNR and other HAT foci in eastern Africa.

Among trypanosomes responsible for AAT, this study identified *T*. *vivax* as the most prevalent species, followed by *T*. *congolense* and *T*. *brucei brucei*. Our findings are congruent with previous findings within the East African savannah [60,61]. The higher numbers of flies with *T*. *vivax* DNA may be due to differences in development cycles in tsetse flies; *T*. *vivax* has all its development stages in the fly’s proboscis unlike *T*. *congolense* and *T*. *brucei*, which establish in the fly midgut where they are affected by low pH, proteases, and lectins [62,63]. Moreover, *T*. *vivax* usually achieves higher parasitemia in hosts than do *T*. *congolense* and *T*. *brucei*, further increasing its chances of being transmitted to tsetse flies during blood-feeding on infected hosts [63]. It is worth noting that this was a cross-sectional study in a fast-changing ecosystem and thus forms the basis for further investigation into effects of seasons, vegetation, and other factors on the prevalence of trypanosomes species. This study used PCR-based methods to determine presence in tsetse fly species, which best detect trypanosome DNA rather than infection status.

The greater abundance of *G*. *pallidipes* but higher rate of trypanosome DNA detected in *G*. *swynnertoni* in the MMNR highlights the need for understanding the difference in susceptibility between the two tsetse species. Differences in susceptibility to trypanosome infection among *Glossina* species has been postulated to be due to the different capabilities of tsetse species-specific mutualistic *Wigglesworthia* bacteria to synthesize folate in their different host species [23]. Vector susceptibility of *G*. *pallidipes* to midgut trypanosomes has been shown to be lower compared to *G*. *morsitans morsitans* and *G*. *morsitans centralis* [64,65]. Further still, tsetse protection against trypanosome invasion has been shown to be different for *G*. *pallidipes* and *G*. *morsitans morsitans* [65]. Similarly, field studies have shown *G*. *swynnertoni* to be more susceptible than *G*. *pallidipes* [54,58]. Given that *G*. *swynnertoni* is an important species in the Maasai Mara-Serengeti ecosystem, its potentially greater susceptibility to trypanosome infection needs further investigation to elucidate its role in trypanosome transmission relative to the more abundant sympatric *G*. *pallidipes*.

Blood feeding of tsetse fly populations in the wild is influenced by the composition of vertebrate host species in an area and how these species attract tsetse flies [12]. Our identification of animal trypanosome DNA in flies with hippopotamus and African buffalo blood-meals was not surprising as these vertebrates are known to be reservoirs for *T*. *vivax*, *T*. *congolense*, and *T*. *brucei* [12,64]. Nevertheless, our findings suggest that animal trypanosomiasis is actively transmitted in this wildlife-livestock interface and may be maintained by multiple potential vertebrate hosts. Despite the abundance of wildebeest, zebra, and other antelopes when the study was conducted (during the Great Migration season), no blood-meals from these hosts were detected in the tsetse flies. This finding is congruent with previous reports that *G*. *pallidipes* and *G*. *swynnertoni* exhibit significant specificity in host selection; wildebeest are not preferred blood-meal sources [13,66] and zebra skin odors are repellant to *G*. *pallidipes* [67]. This study also showed that *G*. *pallidipes* and *G*. *swynnertoni* share vertebrate blood-meal host species. This can be attributed to the fact that the two tsetse species belong to the morsitans group *Glossina*, possibly exhibiting similar host preferences. The influx of people into the MMNR due to heightened tourism during the Great Migration season, may partially explain why humans were frequent blood-meal sources. Nevertheless, identification of mixed blood-meals from humans and wildlife is indicative of the inherent risk of HAT transmission in the MMNR [12,68], even though *T*. *b*. *rhodensiense* was not detected in this study.

Visual cues and odors released by vertebrate hosts influence tsetse fly host choice and have been pivotal to the development of baited traps and targets for the control and management of tsetse fly populations, HAT, and AAT. A tsetse repellant formulation mimicking the odor of waterbuck (*Kobus ellipsiprymnus defassa*), a non-host animal, was recently developed and used as an innovative collar device to protect cattle from tsetse bites and AAT [69]. Visual cues have been extensively exploited in the development of improved traps–stationery and mobile targets impregnated with insecticides for riverine/“palpalis” [70–72] and savannah/“morsitans” [58,73] groups of tsetse. However, for the morsitans group of tsetse flies, including *G*. *pallidipes* and *G*. *swynnertoni*, host odors play a more significant role than visual cues as they strongly attract the tsetse flies across long ranges of up to 100 m [74]. Acetone and butanone odors obtained from cattle have long been used as attractants of choice in tsetse fly control [75]. However, other better tsetse fly attractants, such as 2-propanol, have been identified [76].

Despite this study being cross-sectional, tsetse flies were collected in a season with a high influx of vertebrate hosts into the ecosystem, providing a wide range of choice for host blood-meals. Therefore, our observed high rates of buffalo, hippopotamus, and human blood-meals imply that semiochemicals from these vertebrates may be possible candidates to advance research for novel host-derived cues for controlling *G*. *pallidipes* and *G*. *swynnertoni* populations. This can contribute to existing knowledge on emergent repellant odors and host attractants (such as those described from zebra and waterbuck) [67,69], presenting a unique opportunity to further improve tsetse bait technology. Improved bait technologies could include exploring “Push-Pull” and/or “Attract-and-Kill” approaches. Push-Pull refers to using odorants with a repelling effect that push the arthropods away from the source, thereby protecting animals from their bites and possible disease transmission [77]. Attract-and-Kill refers to use of odorants that attract the arthropods to a target that is treated with an insecticide, thus killing them [77].

Our finding that higher proportions of tsetse flies with *Sodalis* endosymbionts had trypanosome DNA than those without *Sodalis* corroborates previous findings in both wild-caught [25,49] and lab-reared [24,26] tsetse flies. The prevalence of *Sodalis* in this study was lower (6.6%) than the 15.9% prevalence recorded in the Shimba Hills National Reserve, a wildlife-human-livestock interface on Kenya’s south coast [52]. This difference in prevalence may be due to the difference in the locales and study designs, as the previous study was based on a longitudinal survey. Understanding of the functional role of *S*. *glossinidius* in tsetse flies remains limited [21] and was not explored in this study. However, inhibition of tsetse midgut and mouthpart lectins by N-acetyl-D-glucosamine, a product of chitin catabolism by *S*. *glossinidius*, has been proposed as the main factor associated with *S*. *glossinidius* and increased tsetse-vector competence [24,30,78]. Nevertheless, this association is complex as a number of other factors, including geographic location, tsetse fly species, sex, and age also affect the capacity of *S*. *glossinidius* to increase vector competence in wild-caught tsetse flies [30]. While more studies are needed to elucidate the role of *Sodalis* endosymbionts on tsetse competence to vector trypanosomes, our findings suggest that *S*. *glossinidius* symbionts increase the probability of savannah tsetse flies to acquire animal trypanosome infections in this wildlife-livestock interface. In addition to *S*. *glossinidius*, the presence of *Spiroplasma* and *Wolbachia* in tsetse flies in the MMNR presents the region as a favorable site for understanding their potential influence on tsetse vector competence, given that *Spiroplasma* has been shown to reduce tsetse vector competence [19] while *Wolbachia* induces cytoplasmic incompatibility, reducing mating and reproduction capabilities of tsetse flies [79].

This study highlights the sensitivity of HRM analysis to accurately, reliably, rapidly, and reproducibly identify arthropod blood-meal hosts. We were able to identify blood-meals from wild-caught non-engorged flies and detect mixed blood-meals that were confirmed by DNA sequencing. Unlike serological and other PCR-based techniques for blood-meal identification [66,80,81], the use of HRM to detect sequence variants is fast, cost-effective, accurate, easy-to-use, and sensitive, making it a more economical tool for blood-meal analysis [44,46,82,83].

## Conclusions

Emergence and/or reemergence of AT, especially in human-wildlife-interfaces like the MMNR where AT has been recently reported, happens occasionally. With limitations on current methods of control and management of AT and its tsetse fly vectors, more research on the factors influencing trypanosome transmission is required. This study indicates complex interactions of tsetse flies with vertebrate hosts and endosymbionts that may influence maintenance and transmission of African trypanosomes. Our identification of trypanosome DNA in tsetse flies that had fed on hippopotamus and African buffalo highlights these two vertebrate species as possible reservoirs of trypanosomes in the MMNR, providing a basis for investigating their contributions to AT in the MMNR and other wildlife ecosystems. Further understanding of the attractiveness of hippopotamus and expounding existing knowledge on African buffalo attractiveness to tsetse flies based on the volatiles they release, may help to improve tsetse baits and repellants. In addition, our findings indicate that the endosymbiont *S*. *glossinidius* may increase tsetse fly susceptibility to trypanosome infection in this endemic ecology. These findings support the idea that *S*. *glossinidius* can be a potential target for vector control [17]. Despite *T*. *b*. *rhodensiense* not being detected, evidence of tsetse flies feeding on humans and previous reports of *T*. *b*. *rhodensiense* in the MMNR warrant continuous surveillance of human African trypanosomes in the MMNR.

## Supporting information

S1 Fig PCR detection of trypanosome species in tsetse flies. **A**. Agarose gel electrophoresis images of representative tsetse fly samples positive for *Trypanozoon*, *T*. *vivax*, and *T*. *congolense* PCR amplicons with ITS BR/CR primers specific for African trypanosomes species. The trypanozoon group were further resolved using primer pairs specific for *T*. *b*. *rhodesiense* and *T*. *b*. *brucei*. **B**. PCR amplification results for detection of *T*. *b*. *rhodesiense* using primers targeting the SRA gene. **C**. PCR amplification results for detection of *T*. *b*. *brucei* using primers targeting the GPI-PLC gene of *T*. *b*. *brucei*. M represents the molecular ladder;–represents negative control; + represents positive control.
(TIFF)

S2 Fig DNA sequence analysis of mixed blood-meals. **A**. Hippopotamus and human mixed blood-meal cytochrome b sequences aligned and edited using Geneious v8.0.1. **B**. Buffalo and human mixed blood-meal cyt b sequences aligned and edited using Geneious v8.0.1. Scientific names and the GenBank accession numbers highlighted in red represent sequences obtained from this study.
(TIF)

S3 Fig PCR detection of endosymbionts in tsetse flies. **A.** Agarose gel electrophoresis image of a representative PCR amplicons of *S*. *glossinidus* DNA. **B.** Agarose gel electrophoresis image of a representative PCR amplicons for *Wolbachia*.
(TIF)

S1 Table Primer list with annealing temperatures. Details of primer sequences and PCR conditions used.
(XLSX)

S2 Table Trypanosome species and host blood-meals among the tsetse fly species in this study. Distribution of trypanosome infections and blood-meals sources in *Glossina pallidipes* and *Glossina swynnertoni* in the Maasai Mara National Reserve, Kenya.
(XLSX)

S3 Table Data URL repository associated with this study. Details of the nucleotide sequences generated in this study and URLs for obtaining their respective accessions in GenBank.
(XLSX)

## References

[1] Simarro P, Franco J, Diarra A, Postigo RJA, Jannin. Diversity of human African trypanosomiasis epidemiological settings requires fine-tuning control strategies to facilitate disease elimination. Res Rep Trop Med. 2013;4:1–6. 10.2147/RRTM.S40157 30100778 PMC6067614

[2] Cecchi G, Paone M, Feldmann U, Vreysen MJ, Diall O, Mattioli RC. Assembling a geospatial database of tsetse-transmitted animal trypanosomosis for Africa. Parasites and Vectors. 2014;7(1):39–48. 10.1186/1756-3305-7-39 24447638 PMC4015763

[3] Bukachi SA, Wandibba S, Nyamongo IK. The socio-economic burden of human African trypanosomiasis and the coping strategies of households in the South Western Kenya foci. PLoS Negl Trop Dis. 2017;11(10):e0006002 10.1371/journal.pntd.0006002 29073144 PMC5675461

[4] Muhanguzi D, Mugenyi A, Bigirwa G, Kamusiime M, Kitibwa A, Akurut GG, et al African animal trypanosomiasis as a constraint to livestock health and production in Karamoja region: A detailed qualitative and quantitative assessment. BMC Vet Res. 2017;13(1):355–67. 10.1186/s12917-017-1285-z 29178951 PMC5702144

[5] Ngari NN, Gamba DO, Olet PA, Zhao W, Paone M, Cecchi G. Developing a national atlas to support the progressive control of tsetse-transmitted animal trypanosomosis in Kenya. Parasit Vectors. 2020;13:286 10.1186/s13071-020-04156-5 32503681 PMC7275614

[6] Büscher P, Cecchi G, Jamonneau V, Priotto G. Human African trypanosomiasis. Lancet. 2017;390(10110):2397–409. 10.1016/S0140-6736(17)31510-6 28673422

[7] Chitanga S, Marcotty T, Namangala B, van den Bossche P, van den Abbeele J, Delespaux V. High prevalence of drug resistance in animal trypanosomes without a history of drug exposure. PLoS Negl Trop Dis. 2011;5(12):e1454 10.1371/journal.pntd.0001454 22206039 PMC3243716

[8] Giordani F, Morrison LJ, Rowan TG, De Koning HP, Barrett MP. The animal trypanosomiases and their chemotherapy: A review. Parasitology. 2016;143(14):1862–89. 10.1017/S0031182016001268 27719692 PMC5142301

[9] Kotlyar S. Recommendations for control of East African sleeping sickness in Uganda. J Glob Infect Dis. 2010;2(1):43–8. 10.4103/0974-777X.59250 20300417 PMC2840965

[10] Meyer A, Holt HR, Selby R, Guitian J. Past and ongoing tsetse and animal trypanosomiasis control operations in five African countries: A systematic Review. PLoS Negl Trop Dis. 2016;10(12):e0005247 10.1371/journal.pntd.0005247 28027299 PMC5222520

[11] Gaithuma A, Yamagishi J, Hayashida K, Kawai N, Namangala B. Blood meal sources and bacterial microbiome diversity in wild- caught tsetse flies. Sci Rep. 2020;10:5005 10.1038/s41598-020-61817-2 32193415 PMC7081217

[12] Nyingilili HS, Malele II, Nkwengulila G. Diversity of blood meal hosts in *Glossina pallidipes* and its role in the epidemiology of trypanosomiasis at a localized area in Serengeti National Park. Imp J Interdiscip Res. 2016;2(11):1694–8.

[13] Auty H, Cleaveland S, Malele I, Masoy J, Lembo T, Bessell P, et al Quantifying heterogeneity in host-vector contact: Tsetse (*Glossina swynnertoni* and *G*. *pallidipes*) host choice in Serengeti National Park, Tanzania. PLOS ONE. 2016;11(10):e0161291 10.1371/journal.pone.0161291 27706167 PMC5051720

[14] Auty H, Anderson NE, Picozzi K, Lembo T, Mubanga J, Hoare R, et al Trypanosome diversity in wildlife species from the Serengeti and Luangwa Valley ecosystems. PLoS Negl Trop Dis. 2012;6(10):e1828 10.1371/journal.pntd.0001828 23094115 PMC3475651

[15] Anderson NE, Mubanga J, Fevre EM, Picozzi K, Eisler MC, Thomas R, et al Characterisation of the wildlife reservoir community for human and animal trypanosomiasis in the Luangwa Valley, Zambia. PLoS Negl Trop Dis. 2011;5(6):e1211 10.1371/journal.pntd.0001211 21713019 PMC3119639

[16] Herren JK, Mbaisi L, Mararo E, Makhulu EE, Mobegi VA, Butungi H, et al A microsporidian impairs *Plasmodium falciparum* transmission in *Anopheles arabiensis* mosquitoes. Nat Commun. 2020;11:2187 10.1038/s41467-020-16121-y 32366903 PMC7198529

[17] Geiger A, Ponton F, Simo G. Adult blood-feeding tsetse flies, trypanosomes, microbiota and the fluctuating environment in sub-Saharan Africa. ISME J. 2015;9(7):1496–507. 10.1038/ismej.2014.236 25500509 PMC4478693

[18] Kariithi HM, Meki IK, Schneider DI, De Vooght L, Khamis FM, Geiger A, et al Enhancing vector refractoriness to trypanosome infection: Achievements, challenges and perspectives. BMC Microbiol. 2018;18(Suppl 1):3–15. 10.1186/s12866-018-1280-y 30470182 PMC6251094

[19] Schneider DI, Saarman N, Onyango MG, Hyseni C, Opiro R, Echodu R, et al Spatio-temporal distribution of *Spiroplasma* infections in the tsetse fly (*Glossina fuscipes fuscipes*) in northern Uganda. PLoS Negl Trop Dis. 2019;13(8):e0007340 10.1371/journal.pntd.0007340 31369548 PMC6692048

[20] Weiss BL, Wang J, Maltz MA, Wu Y, Aksoy S. Trypanosome infection establishment in the tsetse fly gut is influenced by microbiome-regulated host immune barriers. PLoS Pathog. 2013;9(4):e1003318 10.1371/journal.ppat.1003318 23637607 PMC3630092

[21] Wang J, Weiss BL, Aksoy S. Tsetse fly microbiota: form and function. Front Cell Infect Microbiol. 2013;3:69 10.3389/fcimb.2013.00069 24195062 PMC3810596

[22] Wamwiri FN, Changasi RE. Tsetse flies (*Glossina*) as vectors of human African trypanosomiasis: A review. Biomed Res Int. 2016;2016(Article ID 6201350):8 10.1155/2016/6201350 27034944 PMC4789378

[23] Rio RVM, Jozwick AKS, Savage AF, Sabet A, Vigneron A, Wu Y, et al Mutualist-provisioned resources impact vector competency. MBio. 2019;10:e00018–19. 10.1128/mBio.00018-19 31164458 PMC6550517

[24] Dale C, Welburn SC. The endosymbionts of tsetse flies: Manipulating host-parasite interactions. Int J Parasitol. 2001;31(5–6):628–31. 10.1016/s0020-7519(01)00151-5 11334953

[25] Farikou O, Njiokou F, Mbida Mbida JA, Njitchouang GR, Djeunga HN, Asonganyi T, et al Tripartite interactions between tsetse flies, *Sodalis glossinidius* and trypanosomes-An epidemiological approach in two historical human African trypanosomiasis foci in Cameroon. Infect Genet Evol. 2010;10(1):115–21. 10.1016/j.meegid.2009.10.008 19879380

[26] Wamwiri FN, Ndungu K, Thande PC, Thungu DK, Auma JE, Ngure RM, et al Infection with the secondary tsetse-endosymbiont *Sodalis glossinidius* (Enterobacteriales: *Enterobacteriaceae*) influences parasitism in *Glossina pallidipes* (Diptera: *Glossinidae*). J Insect Sci. 2014;14:272 10.1093/jisesa/ieu134 25527583 PMC5657924

[27] Geiger A, Ravel S, Frutos R, Cuny G. Sodalis glossinidius (*Enterobacteriaceae*) and vectorial competence of *Glossina palpalis gambiensis* and *Glossina morsitans morsitans* for *Trypanosoma congolense Savannah* type. Curr Microbiol. 2005;51(1):35–40. 10.1007/s00284-005-4525-6 15942697

[28] Kame-Ngasse GI, Njiokou F, Melachio-Tanekou TT, Farikou O, Simo G, Geiger A. Prevalence of symbionts and trypanosome infections in tsetse flies of two villages of the “Faro and Déo” division of the Adamawa Region of Cameroon. BMC microbiology. 2018;18(Supp 1):83–91. 30470177 10.1186/s12866-018-1286-5 PMC6251084

[29] Kanté ST, Melachio T, Ofon E, Njiokou F, Simo G. Detection of *Wolbachia* and different trypanosome species in *Glossina palpalis palpalis* populations from three sleeping sickness foci of southern Cameroon. Parasit Vectors. 2018;11(630):1–10. 10.1186/s13071-018-3229-2 30541614 PMC6292098

[30] Channumsin M, Ciosi M, Masiga D, Turner CMR, Mable BK. *Sodalis glossinidius* presence in wild tsetse is only associated with presence of trypanosomes in complex interactions with other tsetse-specific factors. BMC Microbiol. 2018;18(1):163 10.1186/s12866-018-1285-6 30470184 PMC6251152

[31] Rio RVM, Hu Y, Aksoy S. Strategies of the home-team: Symbioses exploited for vector-borne disease control. Trends Microbiol. 2004;12(7):325–36. 10.1016/j.tim.2004.05.001 15223060

[32] Clerinx J, Vlieghe E, Asselman V, van de Casteele S, Maes MB, Lejon V. Human African trypanosomiasis in a Belgian traveller returning from the Masai Mara area, Kenya, February 2012. Eurosurveillance. 2012;17(10):4–7. 22433595

[33] Wolf T, Wichelhaus T, Göttig S, Kleine C, Brodt HR, Just-Nuebling G. *Trypanosoma brucei rhodesiense* infection in a German traveller returning from the Masai Mara area, Kenya, January 2012. Eurosurveillance. 2012;17(10):2–4. 22433594

[34] World Resources Institute. Kenya GIS data. [cited 2020 Aug 21]. Available from: https://www.wri.org/resources/data-sets/kenya-gis-data.

[35] Code for Kenya. Kenya counties shape file. openAfrica; [cited 2020 Aug 21]. Available from: https://africaopendata.org/dataset/kenya-counties-shapefile.

[36] RCMRD GeoPortal. Kenya Admin Boundary Level 0. [cited 2020 Aug 22]. Available from: http://geoportal.rcmrd.org/layers/servir%3Akenya_adm0.

[37] Pollock JN. Description and keys for the identification of *Glossina* species In: Training Manual for Tsetse Control Personnel. Rome: FAO; 1982 p. 147–87.

[38] Adams ER, Hamilton PB, Malele II, Gibson WC. The identification, diversity and prevalence of trypanosomes in field caught tsetse in Tanzania using ITS-1 primers and fluorescent fragment length barcoding. Infect Genet Evol. 2008;8(4):439–44. 10.1016/j.meegid.2007.07.013 17826361

[39] Njiru ZK, Constantine CC, Guya S, Crowther J, Kiragu JM, Thompson RCA, et al The use of ITS1 rDNA PCR in detecting pathogenic African trypanosomes. Parasitol Res. 2005;95(3):186–92. 10.1007/s00436-004-1267-5 15619129

[40] Picozzi K, Carrington M, Welburn SC. A multiplex PCR that discriminates between *Trypanosoma brucei brucei* and zoonotic *T*. *b*. *rhodesiense*. Exp Parasitol. 2008;118(1):41–6. 10.1016/j.exppara.2007.05.014 17643434

[41] Masiga DK, Smyth AJ, Hayes P, Bromidge TJ, Gibson WC. Sensitive detection of trypanosomes in tsetse flies by DNA amplification. Int J Parasitol. 1992;22(7):909–18. 10.1016/0020-7519(92)90047-o 1459784

[42] Kearse M, Moir R, Wilson A, Stones-Havas S, Cheung M, Sturrock S, et al Geneious Basic: An integrated and extendable desktop software platform for the organization and analysis of sequence data. Bioinformatics. 2012;28(12):1647–9. 10.1093/bioinformatics/bts199 22543367 PMC3371832

[43] Altschul SF, Gish W, Miller W, Myers EW, Lipman DJ. Basic local alignment search tool. J Mol Biol. 1990;215(3):403–10. 10.1016/S0022-2836(05)80360-2 2231712

[44] Omondi D, Masiga DK, Ajamma YU, Fielding BC, Njoroge L, Villinger J. Unraveling host-vector-arbovirus interactions by two-gene high resolution melting mosquito bloodmeal analysis in a Kenyan wildlife-livestock interface. PLoS One. 2015;10(7):e0134375 10.1371/journal.pone.0134375 26230507 PMC4521840

[45] Ogola E, Villinger J, Mabuka D, Omondi D, Orindi B, Mutunga J, et al Composition of *Anopheles* mosquitoes, their blood-meal hosts, and *Plasmodium falciparum* infection rates in three islands with disparate bed net coverage in Lake Victoria, Kenya. Malar J. 2017;16:360 10.1186/s12936-017-2015-5 28886724 PMC5591540

[46] Ouso DO, Otiende MY, Jeneby M, Oundo JW, Bargul JL, Miller S, et al Three-gene PCR and high-resolution melting analysis for differentiating vertebrate species mitochondrial DNA for forensic and biodiversity research pipelines. Sci Rep. 2020;10:4741. 10.1038/s41598-020-61600-3 PMC7075967 32179808

[47] Snyder AK, Adkins KZ, Rio RVM. Use of the internal transcribed spacer (ITS) regions to examine symbiont divergence and as a diagnostic tool for Sodalis-related bacteria. Insects. 2011;2(4):515–31. 10.3390/insects2040515 26467831 PMC4553445

[48] Werren JH, Windsor DM. *Wolbachia* infection frequencies in insects: Evidence of a global equilibrium? Proc R Soc B Biol Sci. 2000;267(1450):1277–85. 10.1098/rspb.2000.1139 10972121 PMC1690679

[49] Abd-Alla AMM, Salem TZ, Parker AG, Wang Y, Jehle JA, Vreysen MJB, et al Universal primers for rapid detection of hytrosaviruses. J Virol Methods. 2011;171(1):280–3. 10.1016/j.jviromet.2010.09.025 20923688

[50] Chepkemoi ST, Mararo E, Butungi H, Paredes J, Masiga D, Sinkins SP, et al Identification of *Spiroplasma insolitum* symbionts in *Anopheles gambiae*. Wellcome Open Res. 2017;2:90 10.12688/wellcomeopenres.12468.1 29152597 PMC5668936

[51] Azambuja P, Garcia ES, Ratcliffe NA. Gut microbiota and parasite transmission by insect vectors. Trends Parasitol. 2005;21(12):568–72. 10.1016/j.pt.2005.09.011 16226491

[52] Wamwiri FN, Alam U, Thande PC, Aksoy E, Ngure RM, Aksoy S, et al *Wolbachia*, *Sodalis* and trypanosome co-infections in natural populations of *Glossina austeni* and *Glossina pallidipes*. Parasit Vectors. 2013;6(1):232 10.1186/1756-3305-6-232 23924682 PMC3751944

[53] Ouma JO, Marquez JG, Krafsur ES. Microgeographical breeding structure of the tsetse fly, *Glossina pallidipes* in south-western Kenya. Med Vet Entomol. 2006;20(1):138–49. 10.1111/j.1365-2915.2006.00609.x 16608498 PMC1450340

[54] Malele II, Kinung’hi SM, Nyingilili HS, Matemba LE, Sahani JK, Mlengeya TDK, et al *Glossina* dynamics in and around the sleeping sickness endemic Serengeti ecosystem of northwestern Tanzania. J Vector Ecol. 2007;32(2):263–8. 10.3376/1081-1710(2007)32[263:gdiaat]2.0.co;2 18260516

[55] Nthiwa DM, Odongo DO, Ochanda H, Khamadi S, Gichimu BM. *Trypanosoma* infection rates in *Glossina* species in Mtito Andei Division, Makueni County, Kenya. J Parasitol Res. 2015;2015:607432 10.1155/2015/607432 26617992 PMC4649094

[56] Okeyo WA, Saarman NP, Bateta R, Dion K, Mengual M, Mireji PO, et al Genetic differentiation of *Glossina pallidipes* tsetse flies in Southern Kenya. Am J Trop Med Hyg. 2018;99(4):945–53. 10.4269/ajtmh.18-0154 30105964 PMC6159567

[57] Bateta R, Saarman NP, Okeyo WA, Dion K, Johnson T, Mireji PO, et al Phylogeography and population structure of the tsetse fly *Glossina pallidipes* in Kenya and the serengeti ecosystem. PLoS Negl Trop Dis. 2020;14(2):1–26. 10.1371/journal.pntd.0007855 32092056 PMC7058365

[58] Nagagi YP, Silayo RS, Kweka EJ. Advancements in bait technology to control *Glossina swynnertoni* Austen, the species of limited distribution in Kenya and Tanzania border: A review. J Vector Borne Dis. 2017;54(1):16–24. 28352042

[59] Gobbi F, Bisoffi Z. Human African trypanosomiasis in travellers to Kenya. Eurosurveillance. 2012;17(10):20109 22433593

[60] Simwango M, Ngonyoka A, Nnko HJ, Salekwa LP, Ole-Neselle M, Kimera SI, et al Molecular prevalence of trypanosome infections in cattle and tsetse flies in the Maasai Steppe, northern Tanzania. Parasit Vectors. 2017;10:507 10.1186/s13071-017-2411-2 29061160 PMC5654092

[61] Ngonyoka A, Gwakisa PS, Estes AB, Salekwa LP, Nnko HJ, Hudson PJ, et al Patterns of tsetse abundance and trypanosome infection rates among habitats of surveyed villages in Maasai steppe of northern Tanzania. Infect Dis Poverty. 2017;6(126):1–16. 10.1186/s40249-017-0340-0 28866983 PMC5582388

[62] Rotureau B, Van Den Abbeele J. Through the dark continent: African trypanosome development in the tsetse fly. Front Cell Infect Microbiol. 2013;3:53 10.3389/fcimb.2013.00053 24066283 PMC3776139

[63] Dyer NA, Rose C, Ejeh NO, Acosta-Serrano A. Flying tryps: Survival and maturation of trypanosomes in tsetse flies. Trends Parasitol. 2013;29(4):188–96. 10.1016/j.pt.2013.02.003 23507033

[64] Peacock L, Ferris V, Bailey M, Gibson W. The influence of sex and fly species on the development of trypanosomes in tsetse flies. PLoS Negl Trop Dis. 2012;6(2):e1515 10.1371/journal.pntd.0001515 22348165 PMC3279344

[65] Moloo SK, Sabwa CL, Kabata JM. Vector competence of *Glossina pallidipes* and *G*. *morsitans centralis* for *Trypanosoma vivax*, *T*. *congolense* and *T*. *b*. *brucei*. Acta Trop. 1992;51(3–4):271–80. 10.1016/0001-706x(92)90045-y 1359753

[66] Clausen PH, Adeyemi I, Bauer B, Breloeer M, Salchow F, Staak C. Host preferences of tsetse (Diptera: Glossinidae) based on bloodmeal identifications. Med Vet Entomol. 1998;12(2):169–80. 10.1046/j.1365-2915.1998.00097.x 9622371

[67] Olaide OY, Tchouassi DP, Yusuf AA, Pirk CWW, Masiga DK, Saini RK, et al Zebra skin odor repels the savannah tsetse fly, *Glossina pallidipes* (Diptera: Glossinidae). PLoS Negl Trop Dis. 2019;13(6):e0007460 10.1371/journal.pntd.0007460 31181060 PMC6586361

[68] Wambwa E. Diseases of Importance at the wildlife-livestock interface in Kenya In: Osofsky SA (Ed), Conservation and development interventions at the wildlife/livestock interface: Implications for wildlife, livestock and human health. Gland: IUCN Species Survival Commission; 2005 p. 21–5.

[69] Saini RK, Orindi BO, Mbahin N, Andoke JA, Muasa PN, Mbuvi DM, et al Protecting cows in small holder farms in East Africa from tsetse flies by mimicking the odor profile of a non-host bovid. PLoS Negl Trop Dis. 2017;11(10):e0005977 10.1371/journal.pntd.0005977 29040267 PMC5659797

[70] Lindh JM, Torr SJ, Vale GA, Lehane MJ. Improving the cost-effectiveness of artificial visual baits for controlling the tsetse fly *Glossina fuscipes fuscipes*. PLoS Negl Trop Dis. 2009;3(7):e474 10.1371/journal.pntd.0000474 19582138 PMC2699553

[71] Rayaisse JB, Esterhuizen J, Tirados I, Kaba D, Salou E, Diarrassouba A, et al Towards an optimal design of target for tsetse control: Comparisons of novel targets for the control of palpalis group tsetse in West Africa. PLoS Negl Trop Dis. 2011;5(9):e1332 10.1371/journal.pntd.0001332 21949896 PMC3176748

[72] Kaba D, Zacarie T, M’Pondi AM, Njiokou F, Bosson-Vanga H, Kröber T, et al Standardising visual control devices for tsetse flies: Central and West African species *Glossina palpalis palpalis*. PLoS Negl Trop Dis. 2014;8(1):e2601 10.1371/journal.pntd.0002601 24421909 PMC3888452

[73] Byamungu M, Zacarie T, Makumyaviri M’Pondi A, Mansinsa Diabakana P, McMullin A, Kröber T, et al Standardising visual control devices for tsetse: East and Central African Savannah species *Glossina swynnertoni*, *Glossina morsitans centralis* and *Glossina pallidipes*. PLoS Negl Trop Dis. 2018;12(9):e0006831 10.1371/journal.pntd.0006831 30252848 PMC6173441

[74] Groenendijk CA. The behaviour of tsetse flies in an odour plume. Wageningen University; 1996.

[75] Torr SJ, Hall DR, Smith JL. Responses of tsetse flies (Diptera: Glossinidae) to natural and synthetic ox odours. Bull Entomol Res. 1995;85(1):157–66.

[76] Chahda JS, Soni N, Sun JS, Ebrahim SAM, Weiss BL, Carlson JR. The molecular and cellular basis of olfactory response to tsetse fly attractants. PLoS Genet. 2019;15(3):e1008005 10.1371/journal.pgen.1008005 30875383 PMC6420007

[77] Takken W. Push-pull strategies for vector control. Malar J. 2010;9(S2):I16.

[78] Welburn SC, Maudlin I. Tsetse–Trypanosome Interactions: Rites of Passage. Parasitol Today. 1999;15(10):399–403. 10.1016/s0169-4758(99)01512-4 10481151

[79] Alam U, Medlock J, Brelsfoard C, Pais R, Lohs C, Balmand S, et al *Wolbachia* symbiont infections induce strong cytoplasmic incompatibility in the Tsetse fly *Glossina morsitans*. PLoS Pathog. 2011;7(12):e1002415 10.1371/journal.ppat.1002415 22174680 PMC3234226

[80] Meusnier I, Singer GAC, Landry JF, Hickey DA, Hebert PDN, Hajibabaei M. A universal DNA mini-barcode for biodiversity analysis. BMC Genomics. 2008;9(214):1–4. 10.1186/1471-2164-9-214 18474098 PMC2396642

[81] Peña VH, Fernández GJ, Gómez-Palacio AM, Mejía-Jaramillo AM, Cantillo O, Triana-Chávez O. High-resolution melting (HRM) of the cytochrome B gene: A powerful approach to identify blood-meal sources in Chagas disease vectors. PLoS Negl Trop Dis. 2012;6(2):e1530 10.1371/journal.pntd.0001530 22389739 PMC3289613

[82] Oundo JW, Villinger J, Jeneby M, Ong’amo G, Otiende MY, Makhulu EE, et al Pathogens, endosymbionts, and blood-meal sources of host-seeking ticks in the fast-changing Maasai Mara wildlife ecosystem. PLOS ONE. 2020; 15(8): e0228366 10.1371/journal.pone.0228366 32866142 PMC7458302

[83] Musa AA, Muturi MW, Musyoki AM, Ouso DO, Oundo JW, Makhulu EE, et al Arboviruses and blood meal sources in zoophilic mosquitoes at human-wildlife interfaces in Kenya. Vector-Borne Zoonotic Dis. 2020;40(6):444–453. 10.1089/vbz.2019.2563 32155389

