# Supplementary figures and images for: Tsetse blood-meal sources, endosymbionts and trypanosome-associations in the Maasai Mara National Reserve, a wildlife-human-livestock interface
Source: PLoS Negl Trop Dis. 2021 Jan 6;15(1):e0008267. doi: 10.1371/journal.pntd.0008267 (PMC7822626; doi:10.1371/journal.pntd.0008267)

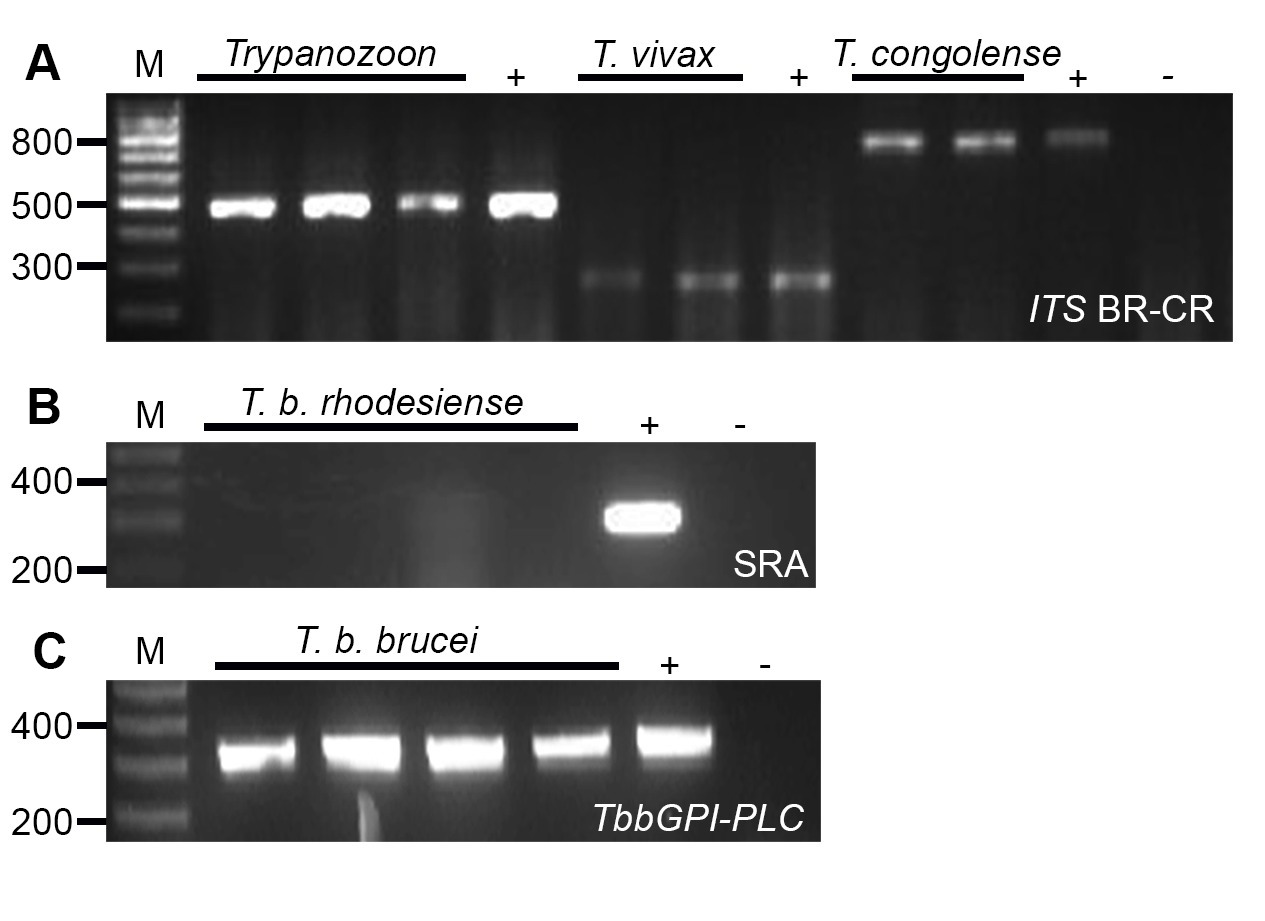

Supplement: S1 Fig — A. Agarose gel electrophoresis images of representative tsetse fly samples positive for Trypanozoon, T. vivax, and T. congolense PCR amplicons with ITS BR/CR primers specific for African trypanosomes species. The trypanozoon group were further resolved using primer pairs specific for T. b. rhodesiense and T. b. brucei. B. PCR amplification results for detection of T. b. rhodesiense using primers targeting the SRA gene. C. PCR amplification results for detection of T. b. brucei using primers targeting the GPI-PLC gene of T. b. brucei. M represents the molecular ladder;–represents negative control; + represents positive control. (TIFF) [file pntd.0008267.s001.tiff]

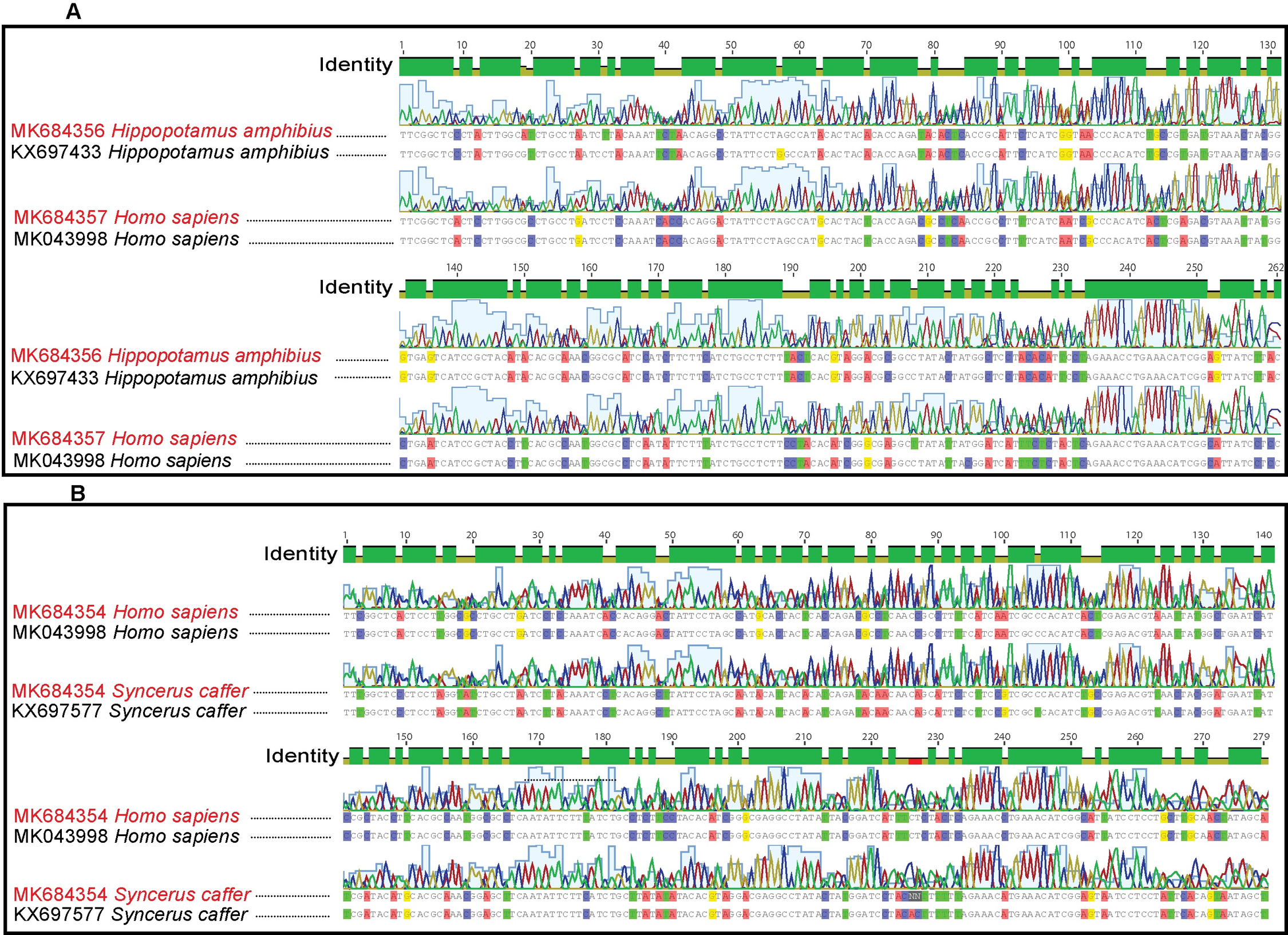

Supplement: S2 Fig — A. Hippopotamus and human mixed blood-meal cytochrome b sequences aligned and edited using Geneious v8.0.1. B. Buffalo and human mixed blood-meal cyt b sequences aligned and edited using Geneious v8.0.1. Scientific names and the GenBank accession numbers highlighted in red represent sequences obtained from this study. (TIF) [file pntd.0008267.s002.tif]

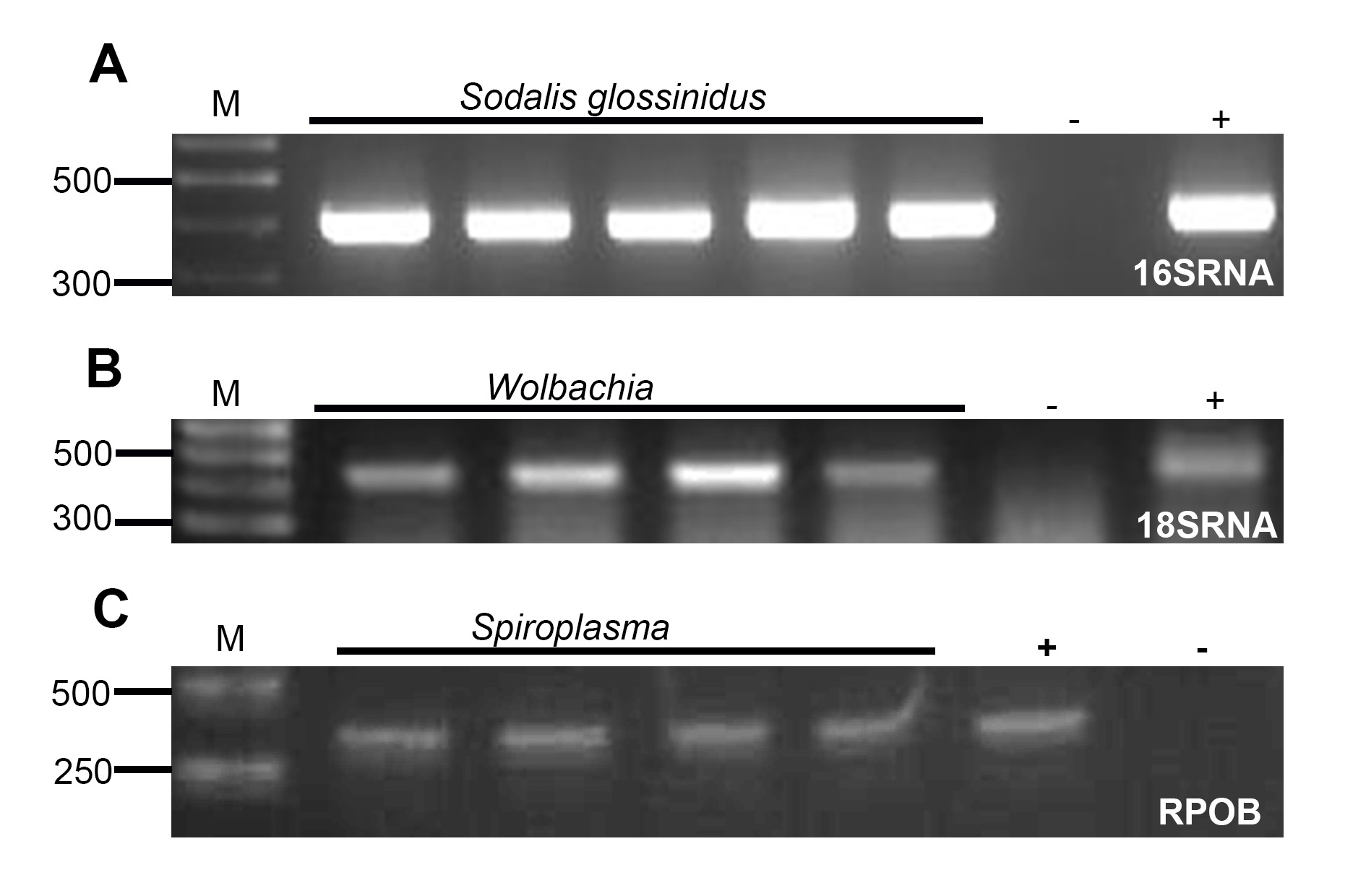

Supplement: S3 Fig — A. Agarose gel electrophoresis image of a representative PCR amplicons of S. glossinidus DNA. B. Agarose gel electrophoresis image of a representative PCR amplicons for Wolbachia. (TIF) [file pntd.0008267.s003.tif]
